# Competitive signaling and cellular communications in myocardial infarction response

**DOI:** 10.1007/s11033-025-10236-5

**Published:** 2025-01-16

**Authors:** Vishnu Nair, Christian Demitri, Finosh G. Thankam

**Affiliations:** 1 Department of Molecular, Cell, & Developmental Biology, University of California, Los Angeles, CA 90095 USA; 2 Department of Experimental Medicine, University of Salento, Lecce, 73100 Italy; 3 Department of Translational Research, College of Osteopathic Medicine of the Pacific, Western University of Health Sciences, Pomona, CA 91766-1854 USA

**Keywords:** Cell communication, Cell competition, Cardiac regeneration, Cardiac healing, Myocardial infarction

## Abstract

Cell communication and competition pathways are malleable to Myocardial Infarction (MI). Key signals, transcriptive regulators, and metabolites associated with apoptotic responses such as Myc, mTOR, and p53 are important players in the myocardium. The individual state of cardiomyocytes, fibroblasts, and macrophages in the heart tissue are adaptable in times of stress. The overlapping communication pathways of Wnt/β-catenin, Notch, and c-Kit exhibit the involvement of important factors in cell competition in the myocardium. Depending on the effects of these pathways on genetic expression and signal amplification, the proliferative capacities of the previously stated cells that make up the myocardium, amplify or diminish. This creates a distinct classification of “fit” and “unfit” cells. Beyond straightforward traits, the intricate metabolite interactions between neighboring cells unveil a complex battle. Strategic manipulation of these pathways holds translational promise for rapid cardiac recovery post-trauma.

## Introduction

Cardiovascular diseases cast a formidable shadow, claiming over 17.6 million lives worldwide [1]. The molecular players and signaling pathways underlying cardiovascular deterioration have been identified; however, their translational significance in human physiology is yet to be achieved. Ischemia of the heart occurs when blood vessels to the myocardium constricts restricting blood flow. Slow recovery from ischemia progresses into myocardial infarction. The cellular mechanisms of acute myocardial infarction follow a pathway of preliminary pro-inflammatory response, cardiomyocyte death, and scar tissue formation. The dynamic response of the myocardium to infarction is regulated by the affected myocardial cells, fibroblasts, and immune cells. As a survival mechanism, growth factors and regenerative/healing signals have been upregulated in surviving myocardial tissue to revive the damage due to ischemia, suggesting communication among the cells within and surrounding the myocardium [2]. However, current treatment approaches targeting the cellular communication signaling for myocardial regeneration are limited. As cardiomyocytes are the primary victims of the infarct heart, they serve as the primary focus for recovery pathophysiology. Common signaling in myocardial cells involves the ligands and receptors of Wnt/β-catenin, Notch, Myc, mTOR, and p53 [3]. These pathways determine the mobilization, proliferation, or differentiation of progenitor cells, as a part of regenerative communications.

Cardiomyocytes (CM) also communicate via autocrine signaling and physical contact using adhesion and gap junctions. The autocrine communication factors released by myocytes include transforming growth factor-β (TGF-β), fibroblast growth factor (FGF), and hepatocyte growth factor (HGF) [4]. These growth factors trigger the production and translation of major cardiac transcription factors/regulators in CM. These transcription factors are involved in the activation of signal transduction pathways, by surface and intracellular receptors, that prime protein kinases to alter specific gene expression and elicit a survival response. Versatile pro-survival proteins are synthesized from modified gene expression, regulating the abundance of CM relative to their growth and apoptotic responses to the proteins [5]. For example, the mitochondrial cytochrome c activates apoptotic proteins and caspases resulting in cell death [6], indicating that cells that proliferate rather than apoptosis have lower cytoplasmic cytochrome c levels. Moreover, connexon proteins of gap junctions permit the firing and spread of action potentials among myocytes given the transfer of ions across membranes [7], which may play a role in timely growth regulatory pathways. Gap junction-induced communication between cardiac fibroblasts (CF) and CM is induced by FGF. Mechanistically, FGF phosphorylates connexon subtypes to accelerate signal transmission via gap junctions [8]. Sustained physical cell connection facilitates the transportation of organelles and myocardial conditioning, permitting myocyte regeneration following injury [9].

Communication pathways are vital for the interaction and response between neighboring cells. For instance, mutant cells are eliminated upon contact with wild-type cells following selective advantage. Hence, less genetically diverse cell populations undergo random replacement events while biased competition poses the possibility for less competent cells to outfit higher competent ones [10]. Therefore, growth rate alone does not determine the competitive ability of a cell, the differences in position within a niche or stem-cell culture conditions are also influencing factors [10]. Current myocardial competition models encompass efficient rivalry among cardiomyocytes and scar tissue forming fibroblasts, however the extent to communication and competition among myocytes, immune cells, and progenitors is yet to be elucidated. Specifically, the common Myc signaling pathway drives myocardial cell competition among cardiomyocytes as the overexpression of c-Myc instills their selective advantage over other myocytes [11]. This is due to the transmission of apoptotic signals by the neighboring cells with lower Myc expression. Since Myc is involved in regulating metabolism, the capacity of cells to manage metabolic activities affects their selective advantage [12]. Similarly, mTOR is necessary for myocardial structural integrity and component cross-movement in protein synthesis and cell proliferation. mTOR signaling is implicated in the innate immune system, cardiac stromal cells, and cardiac fibroblasts during apoptic environments [13]. The tumor suppressor, p53, is also known to maintain healthy cells within the heart and myocardium. Given p53’s remarkable role in cell proliferation suppression, it follows that p53 has been evolutionary conserved among cell phenotypes [14]. It will be apparent that cardiomyocytes and fibroblasts frequently utilize p53 for their mechanistic competition. This intricate dance of signals and competition lays the foundation for the current article, an in-depth exploration of cell communication, competition, and their implications in cardiac regeneration. From the shadows of cardiovascular diseases arises a spotlight on the potential for comprehensive insights and transformative prospects.

## Inflammatory episodes in myocardial infarction

The pro-inflammatory milieu following myocardial injury favors cardiac cell proliferation and regeneration. Tryptophan-kynurenine metabolism contributes to inflammatory responses in blood vessels, prompting CM proliferation [15]. Tryptophan-metabolite enzyme indoleamine-2-3-dioxygenase (IDO) generates tryptophan-kynurenine to activate aryl hydrocarbon receptors (AHR), inducing T-cell and CM differentiation [15]. AHR activation promotes binding to NRF2 promoter regions, triggering the Kynurenine-AHR-NRF2 signaling pathway. NRF2 deficiency damages muscle tissue, especially in skeletal muscles, due to oxidative stress and impaired mitochondrial function. Therefore, maintaining optimal NRF2 levels preserves muscle health [16].

Pro-inflammatory M1 macrophages prime immune responses through phagocytosis and antigen release while anti-inflammatory M2 macrophages stimulate angiogenesis for cardiac repair. Immune cells, engage the wounded site due to inflammatory reactions exhibited by damaged cells or tissue, signaling for tissue healing/regeneration via mobilization, proliferation, and differentiation of other cells that are incorporated within organ structures, thus regaining the lost function. Both regulatory T cells and cardiac B cells, in the immune system, trigger paracrine signaling to induce CM proliferation and regeneration in response to myocardial damage. Following injury, the clearance of cell debris and inhibition of inflammatory signals cardiac B cells initiate renewal signaling pathways. Specifically, the clearance of apoptotic leukocytes increases the prevalence of anti-inflammatory macrophages and reparative cytokines (growth factors) for cardiac repair. Additionally, ligand-activated receptors on cardiac stem cells (CSC) bind cytokine factors, prompting cell proliferation and differentiation [17].

In addition to immune-mediated responses, interference in blood supply (pro-ischemia) to cells in blood vessels and the heart impacts mitochondrial energetics. Stimulation of pro-ischemic injury in stem cells is driven by pro-oxidant pathways that involve the utilization of H_2_O_2_. Consequently, mesenchymal stem cells (MSCs) secrete paracrine factors to promote myocardial stem cell survival in ischemic environments. Cell survival factor, Akt, mitigates CM death in ischemic conditions and phosphorylated Akt delays cell death in ischemic conditions suggesting its protective role [18].

### Cell competition pathways

Cells survive according to their comparative fitness as neighboring competent cells (winners) replace lower competent ones (losers). This competition is intricately linked to metabolic regulation and genomic transcription [10]. Hence, fit cells may be classified as compensatory as their structural and functional characteristics predominate physiological ecosystems. Loser cells are categorized as those that shut down via apoptosis when in contact with compensatory elements of fit cells [19]. Since primitive stages of the life cycle, cell competition ensures that healthy cells thrive to dominate the embryo and thus the organism, over neighboring defective cells [20]. Fitness mechanisms such as Flower-mediated fitness fingerprinting have been developed to demarcate cell fitness in the epithelium, with those containing the wild-type isoform of the Myc-controlled gene [21]. Therefore, qualities such as the abundance of membrane-bound stem cell factors are indicative of hematopoietic stem cell (HSC) competence. Winner HSCs will primarily contribute to blood cell production over time [22]. The chief biomolecular mediators of cell competence, and in turn, cell competition are Myc, mTOR, and p53. Myc is a transcription factor for apoptotic genes, mTOR is a protein kinase that receives signals for growth and proliferation, and p53 is an anti-oncogene that progresses cellular division. Downregulation of Myc is prevalent in loser cells, while its upregulation is advantageous [23]. Increased mTOR signaling encourages growth factor production, promoting the rapid growth and healthy reign of the cells compared to adjacent partners. Additionally, p53 upregulation inhibits cell cycle progression, decreasing the cell competence of those with low levels of p53 [24]. Hence, coordinated and combined regulation of these genes drives the cells that outcompete their locally injured counterparts.

### Wnt/β-catenin pathway

The Wnt signaling pathway releases β-catenin regulated protein growth factors from CSCs to induce cardiogenesis. Wnt signaling transcribes, miR218, which mutes other Wnt antagonists, selectively stimulating cell division and preventing differentiation [25]. Bertozzi et al. detected that the activation of the Wnt/β-catenin pathway accelerated CM regeneration in the heart injury-induced zebrafish model, at the wound site [26]. Wnt/β-catenin signaling pathways are especially apparent in cells on the verge of apoptosis as they increase chances of survival [27]. Uesugi et al. utilized human miRNA mimics to exemplify the mTOR signaling pathway activation by miR218, indicating its link to the Wnt pathway [28]. mTOR promotes the interplay between Wnt components, reducing receptor expression and cell communication, as well as increasing the likelihood of unmonitored cell growth [29]. Wei et al. demonstrated that the upregulation of motor protein, KIF2C, by the Wnt/β-catenin pathway enabled the mTOR pathway, prompting cell maturation in hepatocyte carcinoma patient samples [30]. Fan et al. utilized adult mouse heart and human embryonic stem cell-derived matured CM to enhance myocardial cell development with N-cadherin, increasing the cytoplasmic levels of β-catenin and upregulating Wnt signaling [31]. Cells adhered to each other compete for cytoplasmic β-catenin, meaning the increased Wnt signaling determines their proliferative fate [32]. In addition to the Wnt**/**β-catenin pathway’s role in embryogenesis and cell proliferation, it also dictates Myc expression, allowing its deregulation and cellular abundance [33]. Bisso et al. engineered a mouse model to permit the selective triggering of Myc and Wnt/β-catenin signaling pathways, indicating that stimulation of the Wnt**/**β-catenin pathway increased Myc ability for tumor-like cell proliferation [34].

Neutral competition occurs through random replacement events by equally fit cells, while biased competition dynamics result from mutant cells displaying greater survival over wild types. The Wnt/β-catenin pathway tasked with cell proliferation and division, once left unregulated, creates avenues for biased cell competition. TGF-β pathway and BMP signaling upregulate Bmp 2/7 receptor dimers in mutant cells, consequently increasing differentiation and evolutionary success over wild types [35]. Sturzu et al. ablated mice embryos to distinguish the impact of CM removal on embryonic heart regeneration and found that Wnt/β-catenin signals in cardiac progenitor cells maintained survival and viability to adulthood [36]. Similarly, regulated release of growth factors by the Wnt/β-catenin signaling pathway is stringent on the wild-type form of the p53 coding gene (TP53). As such, a loss of function mutation in TP53 boosts cell growth [37]. Borges et al. demonstrated that mice with p53 loss of function displayed low levels of TP53 along with higher levels of Wnt/β-catenin target genes [38]. Cardiac stress induced by miR-29 via repressing the Wnt signaling pathway was concomitant with a decrease in β-catenin. Sassi et al. demonstrated the role of miR-29 in inhibiting CM proliferation by blocking Wnt pathways, under conditions of cardiac hypertrophy induced by pressure overload [39]. Off-target binding of miRNA to RNA challenges cell competence as repression of target gene expression in biologically fit cells is prevalent [40].

### Notch signaling

**Fig. 1:**
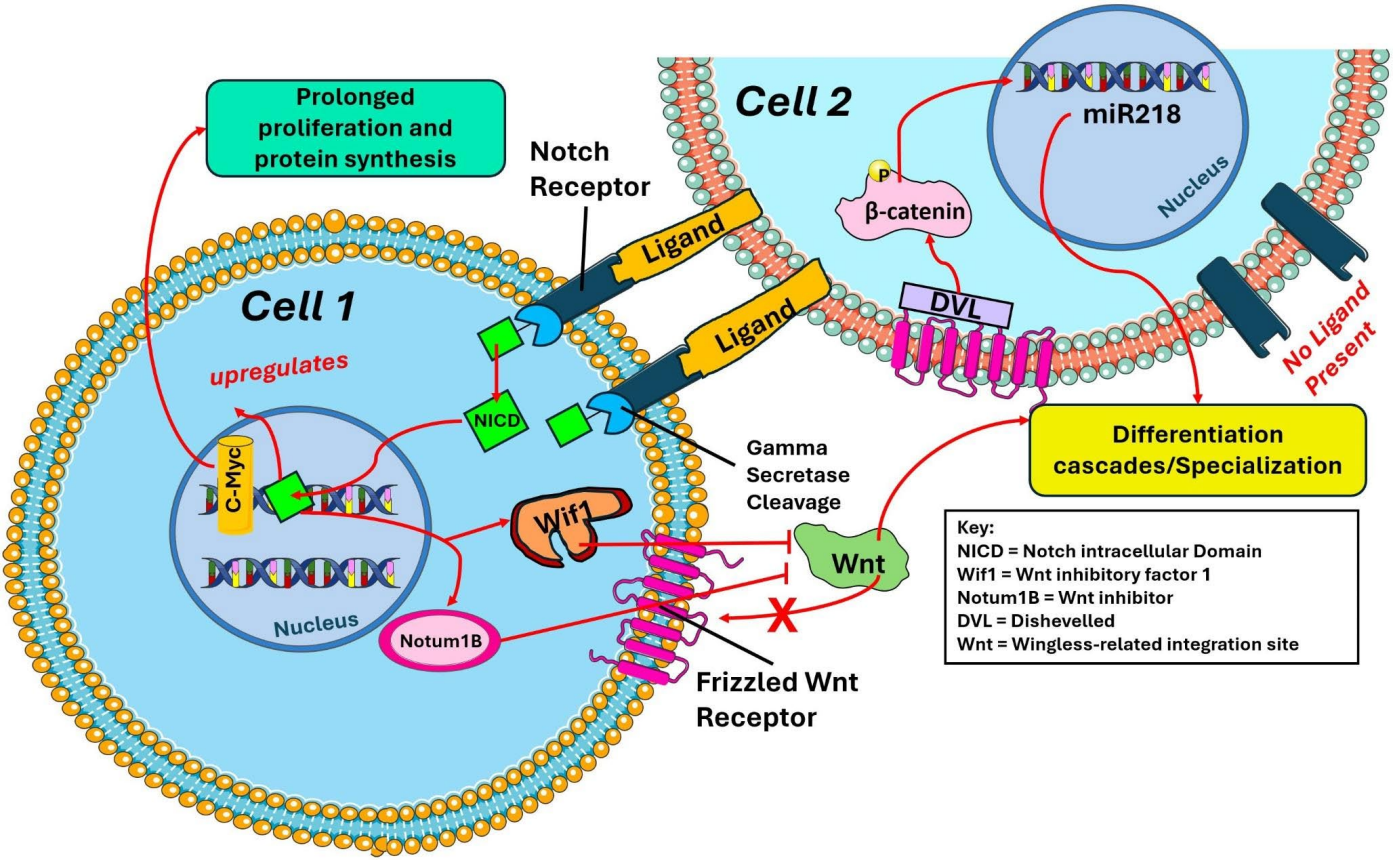
Dynamic Cross-Regulation Between Notch and Wnt/β-Catenin Pathways: The figure depicts two cells, both with Notch and Wnt receptors interacting with each other; cell-1 with Notch activated and cell-2 Wnt/β-Catenin initiated. Ligands from cell-2 attach to the Notch receptors on cell-1 to release the intracellular component of the Notch receptor (NCID). The NCID enters the nucleus to upregulate c-Myc activity and to induce the release of Wnt antagonists Notum1b and Wif1. These components block Wnt activation to its Frizzled receptor. In cell 2, the Wnt attachment to the Frizzled receptor is evident as the protein DVL phosphorylates β-Catenin to induce transcription to miR218. This ultimately differentiates and specializes the cell rather than continued cell growth as seen in cell 1

Notch signaling emerges as a pivotal player in cellular dynamics, particularly in the context of damaged endocardial cells, where its activation promotes regeneration. The cascade begins with ligand-bound Notch receptors breaking down a series of proteins to release cytoplasmic contents that transduce the signal to the nucleus, regulating transcription. Such regulated genes translate to proteins regulating the maturation of specific progenitor cells. When considering the Wnt and Notch intracellular pathways, the inhibition of Notch and activation of Wnt fosters stem cell division and inhibits differentiation of cells. Zhao et al. demonstrated that suppression of Notch signaling in ventricle-damaged zebrafish resulted in minimal fibrosis and muscle regeneration when compared to the control [41]. Further insights into Notch1 signaling come from Palomero et al.‘s work, showcasing its association with c-Myc oncogenes in human T-cells. Knockout of Notch1 downregulates Myc, reducing cell growth and expansion [42]. Herranz et al. collated T-cell hybridization data from lymphoblastic leukemia and established the association of Notch1 in Myc acetylation and unveiled N-Me as a Myc enhancer bound by Notch1 [43]. The reciprocal influence of Wnt and Notch pathways is illustrated in Fig. 1.

Notch signaling becomes a determinant of the probability of cell survival and fitness, especially in the competitive context of cardiac injury. Cardiac injury maintains Notch signaling as Croquelois et al. found that Notch-lacking mice were more susceptible to fibrosis [44]. Pathologically, hypertrophic cells play a crucial role in preventing widespread mortality in myocardial cells, despite the persistence of cardiac complications. Hypertrophy inhibition through Notch signaling, enhances the likelihood of survival compared to cells without Notch expression. This raises questions regarding the relative fitness of hypertrophic and non-hypertrophic cells, as well as those with and without Notch signaling [45].

Chan et al. demonstrated, in human T-cell cell lines, that mTOR protein phosphorylation decreased when the Notch pathway was withdrawn, indicating Notch’s positive control mechanism over mTOR genes [46]. Using adult mice and human organoid cultures, Hibdon et al. depicted how concurrent Notch and mTOR pathway activation significantly increased cell proliferation, and in turn, tumor development [47]. This suggests that overt cell growth, by trumping neighboring cells, is reliant on simultaneously received Notch1 receptor and mTORC1 S6 ribosomal protein target signals. Balaganapathy et al. reported that intracellular components of the Notch domain increase p53 phosphorylation during oxygen and glucose deprivation [48]. Contrastingly, Kim et al. found that Notch signaling downregulated p53 activation in human colon cancer owing to the inhibition of phosphorylation, further preventing the promoter binding ability of p53, and thereby increasing responsive cell growth [49]. Promotion or suppression of the p53 pathway is dependent on Notch signals [50]. Liu et al. reported that senescent muscle stem cell activity in murine models was augmented through Notch and p53 crosstalk [51]. The Notch signaling pathway was found to be activated by regenerative skeletal muscle tissue exhibiting fibroblasts, endothelial cells, and inflammatory cells [52]. The oncogenic nature of p53 has been maneuvered through the Notch pathway, thus enhancing cell proliferation in CMs and replacing wounded scar tissue. Chunhacha et al. found that p53 inhibition in the heart prevented CM apoptosis by comparing failing and non-falling human heart tissue lysates [53]. Given the implications of the Notch pathway, coinciding with p53 activity, it is imperative to various bodily muscle regenerative tactics as their positive potential may translate to cardiomyocytes.

### C-kit receptor pathways

**Fig. 2:**
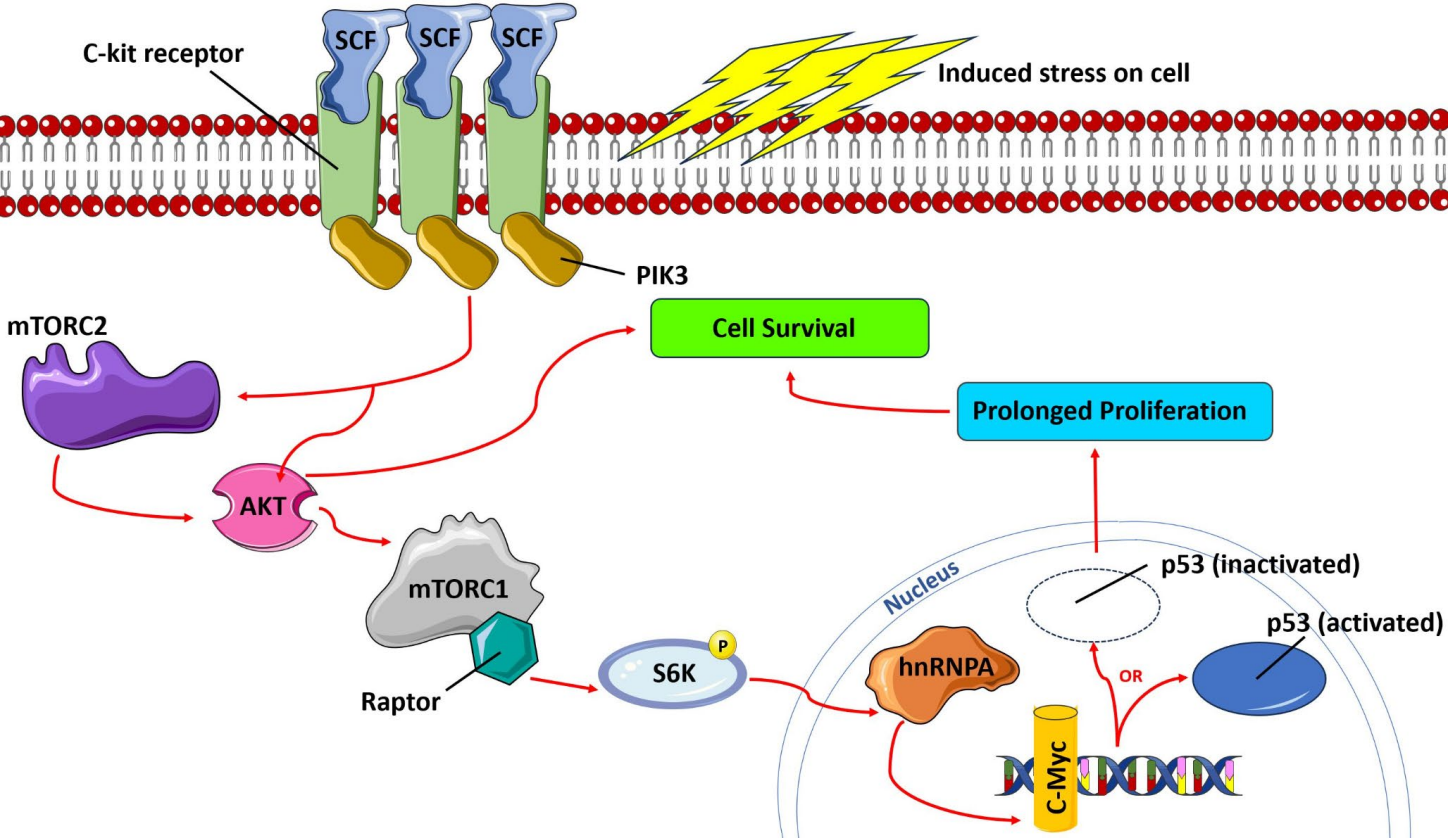
c-Kit Receptor in Signal Cascades Governing Cellular Proliferation: Stem cell factors from neighboring stromal cells and fibroblasts bind the c-kit receptor in stress-induced conditions. Signal transducer, PIK3 activates mTORC2 or phosphorylate AKT to prime the mTORC1 signal pathway. Upon activation, Raptor, an extension of mTORC1, phosphorylates kinase S6K, which triggers hnRNPA to initiate c-Myc transcription. The availability of c-Myc determines the active or inactive state of tumor suppressor p53. p53’s expression accelerates cell proliferation and survival

CSCs are defined by their surface expression of c-kit and stem cell antigens. C-kit is a receptor for stem cell factors and therefore a positive marker for self-renewal. Stem cell factors released by fibroblasts and endothelial cells are important signaling molecules that bind to c-kit. Bound c-kit receptors interact with identical receptor subunits and auto-phosphorylate tyrosine kinases to prime versatile signaling pathways that induce CM generation. This intricate communication activates paracrine feedback between CSCs and CM, responding to myocyte-derived signaling molecules and growth factors [17]. Intercellular signaling binds growth factors to receptors on CSCs, re-prompting independent growth factor production [17]. Siena et al. employed c-kit mutant transgenic mice for the activation of mitogen-activated protein kinase (MAPK) and AKT, improving myocardial remodeling via differentiation of cardiac progenitor cells (CPCs) [54]. Vajravelu et al. used atrial samples to demonstrate that the stimulation of c-kit in CPCs, in the presence of stem cell factor, activates MAPK and PI3K-AKT pathways to modulate the signals that ensure the survival and growth of the injured myocardium. CPCs with gain of function c-kit differentiate into CM, smooth muscle, and endothelial cells more often than those lacking c-kit [55]. In conjunction, Todd et al. reported that the interaction of c-kit ligands with stem cell factors upregulated phosphorylated AKT and activated MAPK/phosphatidylinositol 3-kinase (PI3K), resulting in the development, relocation, and replacement of human melanocytes [56]. Additionally, complete ATK activation demands mTOR stimulation to instill cell survival. Lasota et al. utilized human gastrointestinal stromal cells to illustrate how mTOR mutation eludes c-kit signals [57]. Comparably, Bosken et al. used cultured cells from severely injured patients, unveiling an upregulation of c-kit and downstream mediator CD117 with a concomitant downregulation of phosphorylated mTOR [58]. Overall, c-kit overexpressing stem cells maintain a selective advantage over other cardiac progenitors, as their specific cell lines are conserved. Aquila et al. reported that the activation of c-kit upregulated cardiac progenitors allowed the division and replacement of affected CM in isoproterenol-induced MI mice [59]. Figure 2 depicts how c-kit collaborates with mTOR and intermediate metabolites to maintain a cell’s longevity.

### Myc, mTOR, and p53 signaling pathways

Apoptotic responses emerge as a crucial determinant of the competitive fitness of a cell, which is mainly regulated by mitochondrial activity. Alterations in the expression of mTOR, Myc, and p53 targets induce mitochondrial defects, reducing cell competence. It is important to note that mitochondria play an important role in providing energy as the embryonic heart develops in a hypoxic environment and transitions to an anoxic one. However a shift back to hypoxic conditions as a result of ischemia augments the risk of Reactive Oxygen Species (ROS) induced mitochondrial DNA (mtDNA) damage. MtDNA damage impairs the myocardium’s ability to contract, increasing individual risk for heart failure. Damaged mtDNA signals for inflammatory cell death upon activation of receptors like toll-like receptor 9 (TLR-9) which propagates to neighboring stromal cells [60]. In the context of the myocardium, mtDNA-induced inflammation promotes fibroblast formation of scar tissue. The role of mtDNA as a signaling molecule involved in cell-cell communication revealed that its activation of TLR9 triggers an apoptotic signal cascade [61] which aligns with the effected mitochondria of cardiomyocytes within the infarcted heart. The role of ROS and mtDNA damage on cell-cell communication, specifically competition within the myocardial ecosystem remains widely unknown and should be further pursued with the crossover of the following signaling pathways. Upregulation of p53 and downregulation of mTOR and Myc signals are a feature of loser cells [3]. Ahuja et al. demonstrated that the upregulation of Myc mRNA in the ischemic myocardium of experimental mice is a natural regenerative response to external pressure [62].

Gain of function mutations of Myc and mTOR promotes mutant cell competence, thereby eliminating adjacent wild-type cells (WT), in a process known as Super Competition [63]. Villa del Campo et al. tracked random arrangements of WT cardiac cell populations and augmented Myc cell lineages, indicating a decrease in WT cells and an increase in Myc lines [11]. Concerning stem cells, Minute genes coding for ribosomal proteins displayed reduced differentiation potential and eliminated the loser cells surrounding WTs [63]. Di Giacomo et al. utilized samples of human epithelial tumors to stimulate Myc target genes, portraying that Myc is engaged in caspase-dependent competition with neighboring cells. These observations demonstrate the capacity of Myc for autonomous cellular division and extracellular inhibition [64]. Moreover, the inhibition of mTORC1 primes the stress response during pressure exerted on the myocardium, naturally inducing hypertrophy [65]. Shioi et al. applied mTOR inhibitor, rapamycin, which caused aortic constriction in mice with a concomitant reduction in cardiac hypertrophy [66]. Similarly, Wu et al. utilized MI mice models to inhibit mTORC1 signaling, which exemplified detrimental heart remodeling as evidence of mTOR’s role in cardiac protection/regeneration [67]. Additionally, another component of the mTOR family, mTORC2, provokes adaptations in the myocardium during stress, preventing pressure overload [68].

**Fig. 3:**
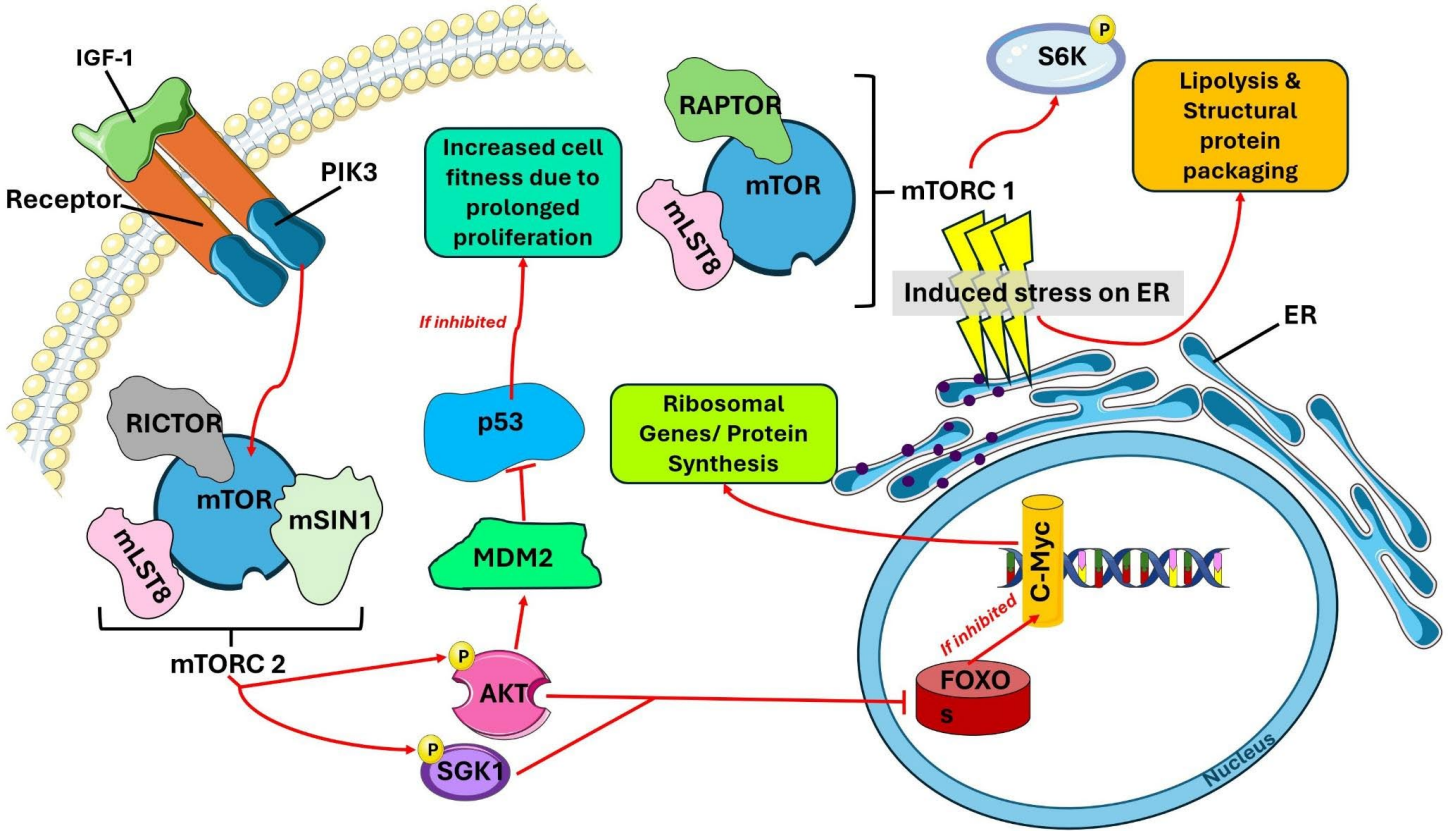
Interplay of Myc, mTOR, and p53 in Cellular Fitness Regulation: Growth factors such as IGF-1 latch onto cell-surface receptors to prime PIK3 where the PIK3 primarily targets mTORC2, making it phosphorylate kinases, AKT, and SGK1. The phosphorylation of these intermediates inhibits FOXOs in the nucleus, which usually act as tumor suppressors. FOXO arrest induces c-Myc transcription, thus supporting ribosomal gene expression and protein synthesis vital to cell cycle interphase. Additionally, AKT and SGK1 phosphorylation causes MDM2 to inhibit p53 tumor suppression, thereby increasing cell proliferative capacity. Meanwhile induced stress on the ER activates the role of mTORC1 in Lipid metabolism and structural development for the cell cycle

Moreover, higher p53 levels are associated with CM hypertrophy. Zhang et al. found that Mdm2 and Mdm4 containing double heterozygous mice cells, with greater p53 activity, were dominated by wildtype cells with lower p53 levels, indicating a slight increase in p53 activity contributes to lower fitness, and susceptibility to cell competition [69]. As Mdm2 is a negative modulator of the p53 gene [70], its presence is a hallmark of fit cardiac cells in infarcted conditions. Shoffner et al. observed that zebrafish had low levels of the protein product of the p53 gene, Tp53, in their injured hearts [71]. Those myocardial cells that maintained lower levels of p53 maintained their competence due to their lack of cell cycle arrest. The intricate interplay among Myc, mTOR, and p53 is rendered in Fig. 3. The signaling pathways that are important to the myocardium and cell fitness are summarized in Table 1.

**Table 1:** The signaling pathways that are important to the myocardium and cell fitness

Pathway	Function	References
Wnt/β-catenin	Cell communication, proliferation, and competition by transcription factor alteration during apoptotic conditions.	[44, 49]
Notch	Determines the transcriptive state of target genes in the cell, controlling stromal cell differentiation by translating to proteins apparent in any differentiated cell type.	[63–65]
C-kit	Uses stem cell factor for angiogenesis, maintaining hematopoietic stem cell lines.	[71]– [72]
Myc	Upregulates transcribed genes for stem cell differentiation and keeps cells in their dormant state until reconditioning signals.	[82]
mTOR	Stimulates muscle stem cell and neural stem cell differentiation through translational genetic regulation and ribosomal interference.	[83]
p53	Determines cell fate, dependent on genetic damage and cellular error, activating or inhibiting apoptotic responses, removing flawed cells, and dominating strong cells.	[86]– [87]

### Cardiac communication and competition at the cellular level

#### Cardiac fibroblasts vs. cardiomyocytes

Fibroblasts and CM engage in intricate communication through paracrine factors, ECM encounters, and electrical, and mechanical interactions. Also, fibroblasts and CM yield cytokines and growth factors that signal to each other for hypertrophic growth in myocytes or fibrosis in the latter. For example, TGF-β1 is present in both cells and influences their respective differentiation capacities. Reperfusion following ischemic conditions is regulated by such cross-signaling between CM and fibroblasts. Forced expression of c-Myc transdifferentiates fibroblasts to CM, suggesting that CM phenotypes allow genealogic factors to transform without transition to stromal phenotypes. In a seminal study by Lyu et al., exosomes from newborn rat cardiac fibroblast were used to record the hypertrophy-inducing signaling factors sent to injured CM. It was concluded that the depletion of CF-derived exosomes minimized CM hypertrophy [72], indicating their mutual association.

Metabolites, such as ATP released from stress-activated CM, stimulate MAPK and p53 activity, converting fibroblasts to myofibroblasts. Mancilla et al. unveiled, with adult p53 loss of function mice, that p53 lacking fibroblasts dispersed more than the wild type as they proliferated greater distances across membranes, creating avenues for contact and competition with additional components of the myocardium [73]. Similarly, miR-133a and miR-30c in CM and cardiac fibroblasts respectively, play a potential role in fibrosis-promoting growth factor release [74]. Initiation of MAPK activity is logically derived from similar mechanisms of c-kit activation, as previously delineated, providing potential for fibroblast growth over myocytes.

Reduced switch from fibroblast to myofibroblast is characteristic of structural changes in mechano-signaling between myocytes and fibroblasts as the heart ages. Mechanosensory proteins identify the affinity of binding between CM and fibroblasts depending on the force the ligand exerts on the receptor. During injury, alterations in motor protein gene expression are phenotypically manifested as a lack of force production, minimized intracellular transmission and cascade, and remolded heart functionality. Fibroblasts maintain the CM-connected ECM through collagen, glycoprotein, and growth factor production. Such stimulatory molecules influence the mechanical mechanisms of CM signaling for hypertrophy, thus associating paracrine signaling with mechanical interaction [75]. Interestingly, myocardial damage triggers the transdifferentiation of cardiac fibroblasts to myofibroblasts via IL-6-driven paracrine signaling and mechanical pressure that risks the structural integrity of the cell [76]. Xiao et al. depicted that cardiac fibroblast differentiation was promoted by upregulating Myc following MI in mice models, provoking apoptosis in adjacent non-fibrotic cells [77]. Myofibroblasts decline as heart tissue injury proceeds to scar in a remodeled ECM which is degraded by early stage cardiac fibroblasts [78]. This dynamic interplay between fibroblasts and CM underlines the multifaceted nature of cardiac tissue response to injury.

### Immune cells vs. cardiomyocytes

During MI, the damaged CM releases alarmins to activate the immune response. Calcagno et al. unveiled that persistent bone marrow-derived neutrophils expressed downstream genes modulated by Myc, indicating its activation during cardiac ischemia [79]. Such a response determines the phagocytic clearance of damaged CM, favoring anti-inflammatory and pro-regenerative responses. Apoptotic cells are detected via macrophage-mediated efferocytosis. The CM debris such as ATP and lipids are released via the pannexin 1 channel to bind with macrophage receptors, suppressing pro-inflammatory cytokine production and enhancing the anti-inflammatory trait. T cell-derived TGF-β returns the cell from an inflammatory state to normal, though CM hypertrophy challenges remodeling events. Dick et al. compared the four functional macrophages in mouse hearts, demonstrating that cardiac tissue lacking C-C chemokine receptor 2 (CCL2) was replaced with CCL2 and major histocompatibility-II (MHCII) [80]. Importantly, the immune cell chemical migration relies on the mTOR pathway targeting CCL2.

Houssari et al. exposed cardiac lymphatic vessels to vascular endothelial growth factor (VEGF) following MI, demonstrating fewer T cell density and proinflammatory macrophages with improved cardiac function [81]. A combination of cell activation and chemically motile signals activate proinflammatory T cells enabling CM to usher more T cells via chemotactic signals. This allows the removal of apoptotic cells by incoming cytotoxic T cells during macrophage penetration. Both CM and immune cells consume higher levels of energy for competition, especially under stressful conditions. mTOR regulates the immune system favoring T cell generation and interaction in the heart [82]. Additionally, CM compete for nutrients and oxygen which induce metabolic signaling with immune cells, or the lack thereof in ischemic conditions, triggering the innate immune response of the heart via interleukin-1 release. T cells negate this response to counteract inflammation through macrophage and fibroblast release. Aside from the blood uptake of macrophages, CM (via connexin 43 gap junctions) are depolarized by the negative resting membrane potential of macrophages, allowing AV node stimulation and heart conduction [83].

### Cardiac fibroblasts vs. immune cells

Hypoxia in the heart triggers the activation of immune cells and cardiac fibroblasts to eliminate and replace damage with scar tissue. T cells directly activate fibrosis through cardiac fibroblasts in the myocardium. Ngwenyama et al. illustrated that helper T cells secreting IFNγ trigger MHCII on cardiac fibroblasts, eliciting positive feedback on helper T cells further accelerating IFNγ secretion and favoring their transition into myofibroblasts [84]. Myofibroblasts are most abundant at wound healing sites, characterized by the immunopositivity of α-smooth muscle actin (αSMA) and collagen type I, and function in ECM repair and reorganization [85]. Additionally, the subsets of T-cells alter the release of versatile growth factors and signals. For example, Th1 increases ECM restructuring while Th2 and Th17 upregulation results in ECM degradation. Similarly, Treg and cytotoxic T-cells maintain collagen homeostasis and decrease collagen synthesis respectively, favoring fibrosis [86]. T cells fail to differentiate when mTOR signaling is repressed as it is needed to incorporate antigen-presenting cell signals within the immune response [87]. mTOR enhances glucose uptake creating an inflammatory environment through further c-Myc expression, signaling for phagocytosis and antigen presentation in immune cells [88].

Fibroblast-derived chemokines and cytokines result in macrophage expansion and T cell abundance, contributing to cardiac remodeling, characterized by the upregulation of collagen I and MMP-mediated ECM destruction by cardiac fibroblasts. Macrophage-derived MMPs heighten the fibrotic response and tissue remodeling through proteolytic communication in the ECM.

### Resident myocardial stromal cells

Stromal cells in the heart maintain cell surface markers and exist in a non-permanent differentiated state. Su et al. cultured human cardiac-derived stromal cells from MI rat and porcine models and displayed angiogenic properties of endothelialized micro-vessels for heart protection [89]. Endothelial cells line the walls of blood vessels; therefore they are subjected to the effects of ischemia and infarction, regulating blood flow to the heart during tissue remodeling. VEGF regulates crosstalk between endothelial and smooth muscle cells of the blood vessels during angiogenesis. During myocardial infarction, coordination between these cell types induces the inflammatory phenotypes noted at onset. Such configurations hope to make up for the lack of oxygen and nutrient supply associated with ischemic conditions. Signaling of resident CSC promotes cell-cell contact for myocardium regeneration. Similarly, Reus et al. demonstrated that cardiac resident stromal cells containing effector proteins such as IL-6, facilitate progenitor progression in the myocardium via growth and differentiation [90]. Rossini et al. demonstrated that injection of cardiac stromal cells and bone marrow mesenchymal stromal cells (MSCs) into infracted rat hearts resulted in up-regulation of adipogenic and osteogenic genes by MSCs whereas cardiac stromal cells upregulated expression of α-SMA and α-Sarc mRNA indicating their differentiation into CM and superior recovery potential [91]. Other resident cells, such as cardiac pericytes that are imperative to angiogenic pathways during infarction, are interior links between macrophages, CF, and CM as their communication is key to ECM synthesis and integrity. Bolli et al. utilized porcine models with coronary occlusion to establish that the resident CSCs revised scarring aftermath and improved LV function [92].

### Recruited stromal cells

MSCs are recruited from adipose and bone marrow to the MI heart for cardiac repair. Bone marrow-derived MSCs differentiate into CM upon the expression of sarcomeric α-actinin and transcription factors by developing gap junctions with resident myocytes. In addition, cardiac hypoxia increases the migration velocity of MSCs, accelerating the repair. Hu et al. found that increased stromal cell-derived factor-1α in ischemic myocardium induced anti-apoptotic kinase activation, accelerating reperfusion by CXCR4 receptors [93]. MSCs release RNAs and surface antigen-containing exosomes as a part of communication between cells within the heart [94]. MSCs respond to apoptotic cell signals from damaged tissue to activate intracellular Fas signaling that leads to proliferation [95]. Cho et al. observed, in mice and rat models of MI, that MSC administration indicated complete myocardial healing owing to MI macrophage switching to M2 phenotype [96]. Hematopoietic stem cells recruited from bone marrow indicated the development of immune cells and circulating blood with endothelial progenitor cells, showing cardiac repair due to capillary population [97].

Upon assessing alpha-actin and smooth muscle content in porcine models receiving bone marrow-derived cells (BMCs), Kim et al. unveiled that BMCs differentiate into endothelial cells and myofibroblasts [98], supporting cardiac healing. Assmus et al. administered bone marrow-derived cells in patients suffering from acute MI, concluding that their uptake in the myocardium elevated the chances of post-trauma survival [99].

Adipose-derived stem cells (ADSCs) have indicated differentiation capacities into CM and vascular smooth muscle cells. As previously delineated, VEGF regulates the angiogenic properties of endothelial and vascular smooth muscle cells to make up for oxygen depleted environments [100]. Nakagami et al. previously unveiled the angiogenic properties of ADSCs as seen through their high expression in CD44 in ischemic mice [101]. Till present, worldwide applications of ADSCs for cardiovascular disorders have primarily improved the capacity of the left ventricle to propel blood. The major communication mediators and their effects on the dominant cells of the myocardium are specified in Table 2.

**Table 2:** The major communication mediators and their effects on the dominant cells of the myocardium

Cell type	Communication mediators used	Mechanisms	References
Cardiomyocytes	Myc, miR-133a, miR-30c, mechanosensory proteins, TGF-β1, alarmins	Hypertrophic growth, transdifferentiation, and ligand-receptor binding affinity.	[93]- [96, 105]
Cardiac Fibroblasts	MAPK, p53, IL-6, Myc, TGF-β1	Stress-induced activation, intracell paracrine signaling, forced cell cycle arrest.	[94, 95, 102]
Immune Cells	C-C chemokine ligand-receptor, TGF-β, mTOR, interleukin-1, IFNγ	Neutrophil activation, proinflammatory T-cell recruitment, and mTOR targeting.	[106, 107]
Myocardial Stromal Cells	IL-6, α-SMA, CXCR4 receptors, M1/M2 macrophages	Progenitor cell progression, Extracellular matrix synthesis, and gap junction development.	

### Cell competition and cardiac regeneration

Cell competition serves as a strategic mechanism to avoid heart failure as CM are swiftly replaced. Manipulation of the intercell signaling pathways promotes competitive capabilities and Myc expression is a key determinant of cell competition. Engineered cells with regulated fitness exposed to the infracted myocardium improve tissue development while eradicating weaker local cells. For instance, Díaz-Díaz et al. reported that mouse embryonic stem cells with diminished Myc levels were apoptotic when surrounded by high-level Myc cells [102]. Unfit cells are continuously removed to prevent mutations and tissue failure which is monitored by Myc upregulation [103]. Also, CSCs induce differentiation of MSCs, forming a cluster of super competitors surrounding the growth deficit of weaker cells. Interestingly, such clusters establish circulatory vesicles for communication, especially in the infarcted heart [104]. Fort et al. treated embryonic stem cells and induced pluripotent stem cells with a WNT pathway activator and later inhibitor to prompt differentiation into CM that self-generated heart contractions. Interestingly, neighboring cells survived apoptosis as a result of this competition [105]. Importantly, inhibiting blood vessel formation during the initial stages of injury allows cardiogenesis to outcompete the formation of scar tissue [106]. Moreover, injured hematopoietic stem and progenitor cells secrete cytokines and growth factors to encourage regrowth in the tissue due to dormant stem cells outcompeting the existing cell line [107]. As described earlier, regulators of apoptosis, namely Myc and Wnt/β-catenin, are the key contributors to cell competition in the myocardium. Such responses to metabolite and cell fate signaling in MI are the attributes of potential pharmacological targets. Lu et al. employed chimeric mice with miRNA155 knockouts to observe wildtype regulatory T cells successfully outcompete the miRNA155 deficient cells. This was attributed to competition for growth factors in lymphocyte-rich blood [108]. Similar comparisons may be drawn to the myocardium as infarcted cells must fight for available growth factors and regulators. With MI-induced porcine models, Williams et al. found that endogenous CSCs in the surviving myocardium are selectively activated while peripheral stem cells on ventricular walls were apoptotic [109]. This demonstrates that post-infarction, there may be regional competition among CSCs from various parts of the heart.

### Translational significance and future

The interconnected role of myocardial components and their possible signaling pathways demonstrates the vast possibilities and profound importance in cardiac tissue regeneration by harnessing the intrinsic growth, cell communication, and competition potential of the cellular components. Interpreting the intricate signals between these components is crucial for developing effective regenerative strategies. A thorough understanding of the activation of these cells under ischemia unveils their translational potential. Wnt/β-catenin, Notch, and C-kit pathways play critical roles in regulating gene expression, cell fate determination, and tissue homeostasis. Further understanding regarding the effects of inhibition/activation of these pathways on target genes and proteins, Myc, mTOR, and p53, would open novel translational insights into the regenerative potential of cells. In addition, the concept of cell competition and protein expression warrants further research. Also, the specific proteins that trigger myofibroblast and CM specialization are of particular importance for the survival and function of the heart. Modulating growth factor signaling between affected cells potentially inhibits the progression of myocardial failure and mitigates symptoms associated with CVD; however, limited information is available warranting further detailed investigations.

Advancements in genome editing technologies, such as CRISPR-Cas9, offer exciting possibilities for cardiac disease modeling and therapy. In the future, these genome editing tools would enable the insertion of activator regions that upregulate gene expression or the deletion of repressors that downregulate within key molecular components promoting translational communications. Epigenetic modifications provide additional avenues for promoting myocardial tissue growth. Additionally, microRNAs (miRNAs) have been identified as key players in post-transcriptional gene regulation, as aforementioned. Understanding the role of such epigenetic modifications in promoting the survival and function of competent CM, fibroblasts, and immune cells would contribute to effective strategies that stabilize heart tissue and restore function following MI. Delving deeper into the mechanisms underlying the regulation of myocardial components, signaling pathways, cell competition, protein expression, genome editing, and epigenetic modifications enhance our translational understanding of cardiac tissue regeneration.

Further research unveiling cell-cell competition and communication is warranted to facilitate the potential therapeutic targets for heart failure paving the way for innovative regenerative approaches to restore and maintain heart health. As we unravel the intricate dance of myocardial components and their signaling symphony, the pursuit of groundbreaking therapeutic strategies becomes not just a scientific endeavor but a promise to mend broken hearts, ushering in an era where cardiac tissue regeneration transcends current boundaries, offering new hope and vitality to those grappling with heart failure.

## Data Availability

No datasets were generated or analysed during the current study.

## References

[1] Benjamin EJ, Muntner P, Alonso A, Bittencourt MS, Callaway CW, Carson AP, Chamberlain AM, Chang AR, Cheng S, Das SR, Delling FN, Djousse L, Elkind MSV, Ferguson JF, Fornage M, Jordan LC, Khan SS, Kissela BM, Knutson KL, Kwan TW, Lackland DT, Lewis TT, Lichtman JH, Longenecker CT, Loop MS, Lutsey PL, Martin SS, Matsushita K, Moran AE, Mussolino ME, O’Flaherty M, Pandey A, Perak AM, Rosamond WD, Roth GA, Sampson UKA, Satou GM, Schroeder EB, Shah SH, Spartano NL, Stokes A, Tirschwell DL, Tsao CW, Turakhia MP, VanWagner LB, Wilkins JT, Wong SS (2019) S.S. Virani, null null, Heart Disease and Stroke Statistics—2019 Update: A Report From the American Heart Association, Circulation 139 e56–e528. 10.1161/CIR.0000000000000659 10.1161/CIR.0000000000000659 30700139

[2] Bayes-Genis A, Roura S, Prat-Vidal C, Farré J, Soler-Botija C, de Luna AB, Cinca J (2007) Chimerism and microchimerism of the human heart: evidence for cardiac regeneration. Nat Rev Cardiol 4:S40–S45. 10.1038/ncpcardio0748 10.1038/ncpcardio0748 17230214

[3] Lima A, Lubatti G, Burgstaller J, Hu D, Green AP, Di Gregorio A, Zawadzki T, Pernaute B, Mahammadov E, Perez-Montero S, Dore M, Sanchez JM, Bowling S, Sancho M, Kolbe T, Karimi MM, Carling D, Jones N, Srinivas S, Scialdone A, Rodriguez TA (2021) Cell competition acts as a purifying selection to eliminate cells with mitochondrial defects during early mouse development. Nat Metab 3:1091–1108. 10.1038/s42255-021-00422-7 34253906 10.1038/s42255-021-00422-7 PMC7611553

[4] Fountoulaki K, Dagres N, Iliodromitis EK (2015) Cellular Communications in the heart. Card Fail Rev 1:64–68. 10.15420/cfr.2015.1.2.64 28785434 10.15420/cfr.2015.1.2.64 PMC5490974

[5] Gotthardt M, Raddatz K (2006) Cardiac Signaling: Cellular, Molecular and clinical aspects. Encyclopedic reference of Genomics and Proteomics in Molecular Medicine. Springer, Berlin, Heidelberg, pp 208–214. 10.1007/3-540-29623-9_4440.

[6] Kang PM, Izumo S (2000) Apoptosis and heart failure. Circul Res 86:1107–1113. 10.1161/01.RES.86.11.1107 10.1161/01.res.86.11.1107 10850960

[7] Kurtenbach S, Kurtenbach S, Zoidl G (2014) Gap junction modulation and its implications for heart function. Front Physiol 5. 10.3389/fphys.2014.00082 10.3389/fphys.2014.00082 PMC3936571 24578694

[8] Liu W, Cui Y, Wei J, Sun J, Zheng L, Xie J (2020) Gap junction-mediated cell-to-cell communication in oral development and oral diseases: a concise review of research progress. Int J Oral Sci 12:1–9. 10.1038/s41368-020-0086-6 32532966 10.1038/s41368-020-0086-6 PMC7293327

[9] Martins-Marques T, Hausenloy DJ, Sluijter JPG, Leybaert L, Girao H (2021) Intercellular Communication in the heart: Therapeutic opportunities for Cardiac Ischemia. Trends Mol Med 27:248–262. 10.1016/j.molmed.2020.10.002 33139169 10.1016/j.molmed.2020.10.002

[10] van Neerven SM, Vermeulen L (2023) Cell competition in development, homeostasis and cancer. Nat Rev Mol Cell Biol 24:221–236. 10.1038/s41580-022-00538-y 36175766 10.1038/s41580-022-00538-y

[11] Villa del Campo C, Clavería C, Sierra R, Torres M (2014) Cell competition promotes phenotypically silent cardiomyocyte replacement in the mammalian heart. Cell Rep 8:1741–1751. 10.1016/j.celrep.2014.08.005 25199831 10.1016/j.celrep.2014.08.005

[12] Esteban-Martínez L, Torres M (2021) Metabolic regulation of cell competition. Dev Biol 475:30–36. 10.1016/j.ydbio.2021.02.011 33652024 10.1016/j.ydbio.2021.02.011

[13] Li X, Yang Z, Nie W, Jiang J, Li S, Li Z, Tian L, Ma X (2019) Exosomes derived from cardiac progenitor cells attenuate CVB3-induced apoptosis via abrogating the proliferation of CVB3 and modulating the mTOR signaling pathways. Cell Death Dis 10:1–16. 10.1038/s41419-019-1910-9 10.1038/s41419-019-1910-9 PMC6751166 31534118

[14] Voskarides K, Giannopoulou N (2023) The role of TP53 in adaptation and evolution. Cells 12:512. 10.3390/cells12030512 36766853 10.3390/cells12030512 PMC9914165

[15] Zhang D, Ning J, Ramprasath T, Yu C, Zheng X, Song P, Xie Z, Zou M-H (2022) Kynurenine promotes neonatal heart regeneration by stimulating cardiomyocyte proliferation and cardiac angiogenesis. Nat Commun 13:6371. 10.1038/s41467-022-33734-7 36289221 10.1038/s41467-022-33734-7 PMC9606021

[16] Huang D-D, Fan S-D, Chen X-Y, Yan X-L, Zhang X-Z, Ma B-W, Yu D-Y, Xiao W-Y, Zhuang C-L, Yu Z (2019) Nrf2 deficiency exacerbates frailty and sarcopenia by impairing skeletal muscle mitochondrial biogenesis and dynamics in an age-dependent manner. Exp Gerontol 119:61–73. 10.1016/j.exger.2019.01.022 30690066 10.1016/j.exger.2019.01.022

[17] Torella D, Ellison GM, Karakikes I, Nadal-Ginard B (2007) Growth-factor-mediated cardiac stem cell activation in myocardial regeneration. Nat Rev Cardiol 4:S46–S51. 10.1038/ncpcardio0772 10.1038/ncpcardio0772 17230215

[18] Miyawaki T, Ofengeim D, Noh K-M, Latuszek-Barrantes A, Hemmings BA, Follenzi A, Zukin RS (2009) The endogenous inhibitor of akt, CTMP, is critical to ischemia-induced neuronal death. Nat Neurosci 12:618–626. 10.1038/nn.2299 19349976 10.1038/nn.2299 PMC2724841

[19] Di Gregorio A, Bowling S, Rodriguez TA (2016) Cell competition and its role in the regulation of cell fitness from Development to Cancer. Dev Cell 38:621–634. 10.1016/j.devcel.2016.08.012 27676435 10.1016/j.devcel.2016.08.012

[20] Bowling S, Di Gregorio A, Sancho M, Pozzi S, Aarts M, Signore M, Schneider MD, Martinez-Barbera JP, Gil J, Rodríguez TA (2018) P53 and mTOR signalling determine fitness selection through cell competition during early mouse embryonic development. Nat Commun 9:1763. 10.1038/s41467-018-04167-y 29720666 10.1038/s41467-018-04167-y PMC5932021

[21] Gogna R, Shee K, Moreno E (2015) Cell competition during growth and regeneration. Annu Rev Genet 49:697–718. 10.1146/annurev-genet-112414-055214 26631518 10.1146/annurev-genet-112414-055214

[22] Miao R, Chun H, Feng X, Gomes AC, Choi J, Pereira JP (2022) Competition between hematopoietic stem and progenitor cells controls hematopoietic stem cell compartment size. Nat Commun 13:4611. 10.1038/s41467-022-32228-w 35941168 10.1038/s41467-022-32228-w PMC9360400

[23] Johnston LA (2014) Socializing with MYC: cell competition in Development and as a model for Premalignant Cancer. Cold Spring Harb Perspect Med 4:a014274. 10.1101/cshperspect.a014274 24692189 10.1101/cshperspect.a014274 PMC3968783

[24] Ozaki T, Nakagawara A (2011) Role of p53 in cell death and human cancers, cancers (Basel) 3. 994–1013. 10.3390/cancers3010994 10.3390/cancers3010994 PMC3756401 24212651

[25] Wang Y, Liu J, Cui J, Sun M, Du W, Chen T, Ming X, Zhang L, Tian J, Li J, Yin L, Liu F, Pu Z, Lv B, Hou J, Yu B (2016) MiR218 modulates wnt signaling in mouse Cardiac Stem cells by promoting proliferation and inhibiting differentiation through a positive feedback Loop. Sci Rep 6:20968. 10.1038/srep20968 26860887 10.1038/srep20968 PMC4748271

[26] Bertozzi A, Wu C-C, Hans S, Brand M, Weidinger G (2022) Wnt/β-catenin signaling acts cell-autonomously to promote cardiomyocyte regeneration in the zebrafish heart. Dev Biol 481:226–237. 10.1016/j.ydbio.2021.11.001 34748730 10.1016/j.ydbio.2021.11.001

[27] Zhang Q, Wang L, Wang S, Cheng H, Xu L, Pei G, Wang Y, Fu C, Jiang Y, He C, Wei Q (2022) Signaling pathways and targeted therapy for myocardial infarction. Sig Transduct Target Ther 7:1–38. 10.1038/s41392-022-00925-z 10.1038/s41392-022-00925-z PMC8913803 35273164

[28] Uesugi A, Kozaki K, Tsuruta T, Furuta M, Morita K, Imoto I, Omura K, Inazawa J (2011) The Tumor suppressive MicroRNA miR-218 targets the mTOR component Rictor and inhibits AKT phosphorylation in oral Cancer. Cancer Res 71:5765–5778. 10.1158/0008-5472.CAN-11-0368 21795477 10.1158/0008-5472.CAN-11-0368

[29] Prossomariti A, Piazzi G, Alquati C, Ricciardiello L (2020) Are Wnt/β-Catenin and PI3K/AKT/mTORC1 distinct pathways in Colorectal Cancer? Cell Mol Gastroenterol Hepatol 10:491–506. 10.1016/j.jcmgh.2020.04.007 32334125 10.1016/j.jcmgh.2020.04.007 PMC7369353

[30] Wei S, Dai M, Zhang C, Teng K, Wang F, Li H, Sun W, Feng Z, Kang T, Guan X, Xu R, Cai M, Xie D (2021) KIF2C: a novel link between Wnt/β-catenin and mTORC1 signaling in the pathogenesis of hepatocellular carcinoma. Protein Cell 12:788–809. 10.1007/s13238-020-00766-y 32748349 10.1007/s13238-020-00766-y PMC8464548

[31] Fan Y, Ho BX, Pang JKS, Pek NMQ, Hor JH, Ng S-Y, Soh B-S (2018) Wnt/β-catenin-mediated signaling re-activates proliferation of matured cardiomyocytes. Stem Cell Res Ther 9:338. 10.1186/s13287-018-1086-8 30526659 10.1186/s13287-018-1086-8 PMC6286613

[32] Piven OO, Winata CL (2017) The canonical way to make a heart: β-catenin and plakoglobin in heart development and remodeling. Exp Biol Med (Maywood) 242:1735–1745. 10.1177/1535370217732737 28920469 10.1177/1535370217732737 PMC5714149

[33] Rennoll S, Yochum G (2015) Regulation of MYC gene expression by aberrant Wnt/β-catenin signaling in colorectal cancer. World J Biol Chem 6:290–300. 10.4331/wjbc.v6.i4.290 26629312 10.4331/wjbc.v6.i4.290 PMC4657124

[34] Bisso A, Filipuzzi M, Gamarra Figueroa GP, Brumana G, Biagioni F, Doni M, Ceccotti G, Tanaskovic N, Morelli MJ, Pendino V, Chiacchiera F, Pasini D, Olivero D, Campaner S, Sabò A, Amati B (2020) Cooperation between MYC and β-Catenin in Liver Tumorigenesis requires Yap/Taz. Hepatology 72:1430–1443. 10.1002/hep.31120 31965581 10.1002/hep.31120

[35] Colozza G, Park S-Y, Koo B-K (2022) Clone wars: from molecules to cell competition in intestinal stem cell homeostasis and disease. Exp Mol Med 54:1367–1378. 10.1038/s12276-022-00854-5 36117218 10.1038/s12276-022-00854-5 PMC9534868

[36] Sturzu AC, Rajarajan K, Passer D, Plonowska K, Riley A, Tan TC, Sharma A, Xu AF, Engels MC, Feistritzer R, Li G, Selig MK, Geissler R, Robertson KD, Scherrer-Crosbie M, Domian IJ, Wu SM (2015) Fetal mammalian heart generates a robust compensatory response to cell loss. Circulation 132:109–121. 10.1161/CIRCULATIONAHA.114.011490 25995316 10.1161/CIRCULATIONAHA.114.011490 PMC4516129

[37] Xiao Q, Werner J, Venkatachalam N, Boonekamp KE, Ebert MP, Zhan T (2022) Cross-talk between p53 and wnt signaling in Cancer. Biomolecules 12:453. 10.3390/biom12030453 35327645 10.3390/biom12030453 PMC8946298

[38] Borges KS, Pignatti E, Leng S, Kariyawasam D, Ruiz-Babot G, Ramalho FS, Taketo MM, Carlone DL, Breault DT (2020) Wnt/β-catenin activation cooperates with loss of p53 to cause adrenocortical carcinoma in mice. Oncogene 39:5282–5291. 10.1038/s41388-020-1358-5 32561853 10.1038/s41388-020-1358-5 PMC7378041

[39] Sassi Y, Avramopoulos P, Ramanujam D, Grüter L, Werfel S, Giosele S, Brunner A-D, Esfandyari D, Papadopoulou AS, De Strooper B, Hübner N, Kumarswamy R, Thum T, Yin X, Mayr M, Laggerbauer B (2017) Engelhardt, Cardiac myocyte miR-29 promotes pathological remodeling of the heart by activating wnt signaling. Nat Commun 8:1614. 10.1038/s41467-017-01737-4 29158499 10.1038/s41467-017-01737-4 PMC5696364

[40] Kilikevicius A, Meister G, Corey DR (2022) Reexamining assumptions about miRNA-guided gene silencing. Nucleic Acids Res 50:617. 10.1093/nar/gkab1256 34967419 10.1093/nar/gkab1256 PMC8789053

[41] Zhao L, Ben-Yair R, Burns CE, Burns CG (2019) Endocardial Notch Signaling promotes Cardiomyocyte Proliferation in the regenerating zebrafish heart through wnt pathway antagonism. Cell Rep 26:546–554e5. 10.1016/j.celrep.2018.12.048 30650349 10.1016/j.celrep.2018.12.048 PMC6366857

[42] Palomero T, Lim WK, Odom DT, Sulis ML, Real PJ, Margolin A, Barnes KC, O’Neil J, Neuberg D, Weng AP, Aster JC, Sigaux F, Soulier J, Look AT, Young RA, Califano A, Ferrando AA (2006) NOTCH1 directly regulates c-MYC and activates a feed-forward-loop transcriptional network promoting leukemic cell growth. Proc Natl Acad Sci 103:18261–18266. 10.1073/pnas.0606108103 17114293 10.1073/pnas.0606108103 PMC1838740

[43] Herranz D, Ambesi-Impiombato A, Palomero T, Schnell SA, Belver L, Wendorff AA, Xu L, Castillo-Martin M, Llobet-Navás D, Cardo CC, Clappier E, Soulier J, Ferrando AA (2014) N-Me, a long range oncogenic enhancer in T-cell acute lymphoblastic leukemia. Nat Med 20:1130–1137. 10.1038/nm.3665 25194570 10.1038/nm.3665 PMC4192073

[44] Croquelois A, Domenighetti AA, Nemir M, Lepore M, Rosenblatt-Velin N, Radtke F, Pedrazzini T (2008) Control of the adaptive response of the heart to stress via the Notch1 receptor pathway. J Exp Med 205:3173–3185. 10.1084/jem.20081427 19064701 10.1084/jem.20081427 PMC2605223

[45] Kachanova O, Lobov A, Malashicheva A (2022) The role of the Notch Signaling Pathway in Recovery of Cardiac function after myocardial infarction. Int J Mol Sci 23:12509. 10.3390/ijms232012509 36293363 10.3390/ijms232012509 PMC9604421

[46] Chan SM, Weng AP, Tibshirani R, Aster JC (2007) Utz1, notch signals positively regulate activity of the mTOR pathway in T-cell acute lymphoblastic leukemia. Blood 110:278–286. 10.1182/blood-2006-08-039883 17363738 10.1182/blood-2006-08-039883 PMC1896117

[47] Hibdon ES, Razumilava N, Keeley TM, Wong G, Solanki S, Shah YM, Samuelson LC (2019) Notch and mTOR Signaling pathways Promote Human gastric Cancer cell proliferation. Neoplasia 21:702–712. 10.1016/j.neo.2019.05.002 31129492 10.1016/j.neo.2019.05.002 PMC6536707

[48] Balaganapathy P, Baik S-H, Mallilankaraman K, Sobey CG, Jo D-G, Arumugam TV (2018) Interplay between Notch and p53 promotes neuronal cell death in ischemic stroke. J Cereb Blood Flow Metab 38:1781–1795. 10.1177/0271678X17715956 28617078 10.1177/0271678X17715956 PMC6168918

[49] Kim SB, Chae GW, Lee J, Park J, Tak H, Chung JH, Park TG, Ahn JK, Joe CO (2007) Activated Notch1 interacts with p53 to inhibit its phosphorylation and transactivation. Cell Death Differ 14:982–991. 10.1038/sj.cdd.4402083 17186020 10.1038/sj.cdd.4402083

[50] Dotto GP (2009) Crosstalk of notch with p53 and p63 in cancer growth control. Nat Rev Cancer 9:587–595. 10.1038/nrc2675 19609265 10.1038/nrc2675 PMC6059364

[51] Liu L, Charville GW, Cheung TH, Yoo B, Santos PJ, Schroeder M, Rando TA (2018) Impaired Notch Signaling leads to a decrease in p53 activity and mitotic catastrophe in aged muscle stem cells. Cell Stem Cell 23:544–556e4. 10.1016/j.stem.2018.08.019 30244867 10.1016/j.stem.2018.08.019 PMC6173623

[52] Gioftsidi S, Relaix F, Mourikis P (2022) The notch signaling network in muscle stem cells during development, homeostasis, and disease. Skelet Muscle 12:9. 10.1186/s13395-022-00293-w 35459219 10.1186/s13395-022-00293-w PMC9027478

[53] Chunhacha P, Pinkaew D, Sinthujaroen P, Bowles DE, Fujise K (2021) Fortilin inhibits p53, halts cardiomyocyte apoptosis, and protects the heart against heart failure. Cell Death Discov 7:1–10. 10.1038/s41420-021-00692-w 10.1038/s41420-021-00692-w PMC8542040 34689154

[54] Di Siena S, Gimmelli R, Nori SL, Barbagallo F, Campolo F, Dolci S, Rossi P, Venneri MA, Giannetta E, Gianfrilli D, Feigenbaum L, Lenzi A, Naro F, Cianflone E, Mancuso T, Torella D, Isidori AM, Pellegrini M (2016) Activated c-Kit receptor in the heart promotes cardiac repair and regeneration after injury. Cell Death Dis 7:e2317–e2317. 10.1038/cddis.2016.205 27468693 10.1038/cddis.2016.205 PMC4973348

[55] Vajravelu BN, Hong KU, Al-Maqtari T, Cao P, Keith MCL, Wysoczynski M, Zhao J, Moore JB, Bolli IVR (2015) C-Kit promotes Growth and Migration of Human Cardiac Progenitor cells via the PI3K-AKT and MEK-ERK pathways. PLoS ONE 10:e0140798. 10.1371/journal.pone.0140798 26474484 10.1371/journal.pone.0140798 PMC4608800

[56] Todd JR, Scurr LL, Becker TM, Kefford RF, Rizos H (2014) The MAPK pathway functions as a redundant survival signal that reinforces the PI3K cascade in c-Kit mutant melanoma. Oncogene 33:236–245. 10.1038/onc.2012.562 23246970 10.1038/onc.2012.562

[57] Lasota J, Kowalik A, Felisiak-Golabek A, Zięba S, Wang Z-F, Miettinen M (2019) New mechanisms of mTOR Pathway activation in KIT-mutant malignant GISTs. Appl Immunohistochem Mol Morphol 27:54–58. 10.1097/PAI.0000000000000541 28777148 10.1097/PAI.0000000000000541 PMC8191381

[58] Bösken B, Hepner-Schefczyk M, Vonderhagen S, Dudda M, Flohé SB (2020) An Inverse Relationship Between c-Kit/CD117 and mTOR Confers NK Cell Dysregulation Late After Severe Injury, Frontiers in Immunology 11 https://www.frontiersin.org/articles/10.3389/fimmu.2020.01200 (accessed June 18, 2023) 10.3389/fimmu.2020.01200 PMC7330140 32670280

[59] Aquila I, Cianflone E, Scalise M, Marino F, Mancuso T, Filardo A, Smith AJ, Cappetta D, De Angelis A, Urbanek K, Isidori AM, Torella M, Agosti V, Viglietto G, Nadal-Ginard B, Ellison-Hughes GM (2019) Torella, c-kit haploinsufficiency impairs adult cardiac stem cell growth, myogenicity and myocardial regeneration. Cell Death Dis 10:1–19. 10.1038/s41419-019-1655-5 10.1038/s41419-019-1655-5 PMC6547756 31164633

[60] Ward GA, Dalton RP, Meyer BS, McLemore AF, Aldrich AL, Lam NB, Onimus AH, Vincelette ND, Trinh TL, Chen X, Calescibetta AR, Christiansen SM, Hou H-A, Johnson JO, Wright KL, Padron E, Eksioglu EA, List AF (2023) Oxidized mitochondrial DNA engages TLR9 to activate the NLRP3 inflammasome in myelodysplastic syndromes. Int J Mol Sci 24:3896. 10.3390/ijms24043896 36835307 10.3390/ijms24043896 PMC9966808

[61] Atarashi N, Morishita M, Matsuda S (2024) Activation of innate immune receptor TLR9 by mitochondrial DNA plays essential roles in the chemical long-term depression of hippocampal neurons. J Biol Chem 300:105744. 10.1016/j.jbc.2024.105744 38354781 10.1016/j.jbc.2024.105744 PMC10943477

[62] Ahuja P, Zhao P, Angelis E, Ruan H, Korge P, Olson A, Wang Y, Jin ES, Jeffrey FM, Portman M, MacLellan WR (2010) Myc controls transcriptional regulation of cardiac metabolism and mitochondrial biogenesis in response to pathological stress in mice. J Clin Invest 120:1494–1505. 10.1172/JCI38331 20364083 10.1172/JCI38331 PMC2860901

[63] Yusupova M, Fuchs Y (2023) To not love thy neighbor: mechanisms of cell competition in stem cells and beyond. Cell Death Differ 30:979–991. 10.1038/s41418-023-01114-3 36813919 10.1038/s41418-023-01114-3 PMC10070350

[64] Di Giacomo S, Sollazzo M, de Biase D, Ragazzi M, Bellosta P, Pession A, Grifoni D (2017) Human Cancer cells Signal their competitive fitness through MYC activity. Sci Rep 7:12568. 10.1038/s41598-017-13002-1 28974715 10.1038/s41598-017-13002-1 PMC5626713

[65] Sciarretta S, Volpe M, Sadoshima J (2014) mTOR Signaling in Cardiac Physiology and Disease. Circ Res 114:549–564. 10.1161/CIRCRESAHA.114.302022 24481845 10.1161/CIRCRESAHA.114.302022 PMC3995130

[66] Shioi T, McMullen JR, Tarnavski O, Converso K, Sherwood MC, Manning WJ, Izumo S (2003) Rapamycin attenuates load-Induced Cardiac Hypertrophy in mice. Circulation 107:1664–1670. 10.1161/01.CIR.0000057979.36322.88 12668503 10.1161/01.CIR.0000057979.36322.88

[67] Wu X, Cao Y, Nie J, Liu H, Lu S, Hu X, Zhu J, Zhao X, Chen J, Chen X, Yang Z, Li X (2013) Genetic and pharmacological inhibition of Rheb1-mTORC1 signaling exerts cardioprotection against adverse cardiac remodeling in mice. Am J Pathol 182:2005–2014. 10.1016/j.ajpath.2013.02.012 23567640 10.1016/j.ajpath.2013.02.012

[68] Sciarretta S, Forte M, Frati G, Sadoshima J (2018) New insights into the role of mTOR Signaling in the Cardiovascular System. Circul Res 122:489–505. 10.1161/CIRCRESAHA.117.311147 10.1161/CIRCRESAHA.117.311147 PMC6398933 29420210

[69] Zhang G, Xie Y, Zhou Y, Xiang C, Chen L, Zhang C, Hou X, Chen J, Zong H, Liu G (2017) p53 pathway is involved in cell competition during mouse embryogenesis. Proc Natl Acad Sci U S A 114:498–503. 10.1073/pnas.1617414114 28049824 10.1073/pnas.1617414114 PMC5255589

[70] Chène P (2003) Inhibiting the p53–MDM2 interaction: an important target for cancer therapy. Nat Rev Cancer 3:102–109. 10.1038/nrc991 12563309 10.1038/nrc991

[71] Shoffner A, Cigliola V, Lee N, Ou J, Poss KD (2020) Tp53 suppression promotes cardiomyocyte proliferation during zebrafish heart regeneration. Cell Rep 32:108089. 10.1016/j.celrep.2020.108089 32877671 10.1016/j.celrep.2020.108089 PMC7494019

[72] Lyu L, Wang H, Li B, Qin Q, Qi L, Nagarkatti M, Nagarkatti P, Janicki JS, Wang XL, Cui T (2015) A critical role of cardiac fibroblast-derived exosomes in activating renin angiotensin system in cardiomyocytes. J Mol Cell Cardiol 89:268–279. 10.1016/j.yjmcc.2015.10.022 26497614 10.1016/j.yjmcc.2015.10.022 PMC4988239

[73] Mancilla TR, Davis LR, Aune GJ (2020) Doxorubicin-induced p53 interferes with mitophagy in cardiac fibroblasts. PLoS ONE 15:e0238856. 10.1371/journal.pone.0238856 32960902 10.1371/journal.pone.0238856 PMC7508395

[74] Bang C, Antoniades C, Antonopoulos AS, Eriksson U, Franssen C, Hamdani N, Lehmann L, Moessinger C, Mongillo M, Muhl L, Speer T, Thum T (2015) Intercellular communication lessons in heart failure. Eur J Heart Fail 17:1091–1103. 10.1002/ejhf.399 26398116 10.1002/ejhf.399

[75] Perbellini F, Watson SA, Bardi I, Terracciano CM (2018) Heterocellularity and Cellular Cross-talk in the Cardiovascular System. Front Cardiovasc Med 5:143. 10.3389/fcvm.2018.00143 30443550 10.3389/fcvm.2018.00143 PMC6221907

[76] Hall C, Gehmlich K, Denning C, Pavlovic D (2021) Complex relationship between cardiac fibroblasts and cardiomyocytes in Health and Disease. J Am Heart Association 10:e019338. 10.1161/JAHA.120.019338 10.1161/JAHA.120.019338 PMC8174279 33586463

[77] Xiao Y, Hill MC, Li L, Deshmukh V, Martin TJ, Wang J, Martin JF (2019) Hippo pathway deletion in adult resting cardiac fibroblasts initiates a cell state transition with spontaneous and self-sustaining fibrosis. Genes Dev 33:1491–1505. 10.1101/gad.329763.119 31558567 10.1101/gad.329763.119 PMC6824468

[78] Chen W, Bian W, Zhou Y, Zhang J, Fibroblasts C, Regeneration M (2021) Frontiers in Bioengineering and Biotechnology 9 https://www.frontiersin.org/articles/10.3389/fbioe.2021.599928 (accessed May 14, 2023) 10.3389/fbioe.2021.599928 PMC8026894 33842440

[79] Calcagno DM, Zhang C, Toomu A, Huang K, Ninh VK, Miyamoto S, Aguirre AD, Fu Z, Heller Brown J, King KR (2021) SiglecF(HI) Marks Late-Stage neutrophils of the Infarcted Heart: a single‐cell transcriptomic analysis of neutrophil diversification. J Am Heart Assoc 10:e019019. 10.1161/JAHA.120.019019 33525909 10.1161/JAHA.120.019019 PMC7955351

[80] Dick SA, Macklin JA, Nejat S, Momen A, Clemente-Casares X, Althagafi MG, Chen J, Kantores C, Hosseinzadeh S, Aronoff L, Wong A, Zaman R, Barbu I, Besla R, Lavine KJ, Razani B, Ginhoux F, Husain M, Cybulsky MI, Robbins CS, Epelman S (2019) Self-renewing resident cardiac macrophages limit adverse remodeling following myocardial infarction. Nat Immunol 20:29–39. 10.1038/s41590-018-0272-2 30538339 10.1038/s41590-018-0272-2 PMC6565365

[81] Houssari M, Dumesnil A, Tardif V, Kivelä R, Pizzinat N, Boukhalfa I, Godefroy D, Schapman D, Hemanthakumar KA, Bizou M, Henry J-P, Renet S, Riou G, Rondeaux J, Anouar Y, Adriouch S, Fraineau S, Alitalo K, Richard V, Mulder P (2020) Brakenhielm, Lymphatic and Immune Cell Cross-talk regulates Cardiac Recovery after experimental myocardial infarction, arteriosclerosis, thrombosis, and Vascular Biology. 40:1722–1737. 10.1161/ATVBAHA.120.314370 10.1161/ATVBAHA.120.314370 PMC7310303 32404007

[82] Xu L, Brink M (2016) mTOR, cardiomyocytes and inflammation in cardiac hypertrophy, Biochimica et Biophysica Acta (BBA) -. Mol Cell Res 1863:1894–1903. 10.1016/j.bbamcr.2016.01.003 10.1016/j.bbamcr.2016.01.003 26775585

[83] Hulsmans M, Clauss S, Xiao L, Aguirre AD, King KR, Hanley A, Hucker WJ, Wülfers EM, Seemann G, Courties G, Iwamoto Y, Sun Y, Savol AJ, Sager HB, Lavine KJ, Fishbein GA, Capen DE, Da Silva N, Miquerol L, Wakimoto H, Seidman CE, Seidman JG, Sadreyev RI, Naxerova K, Mitchell RN, Brown D, Libby P, Weissleder R, Swirski FK, Kohl P, Vinegoni C, Milan DJ, Ellinor PT, Nahrendorf M (2017) Macrophages Facilitate Electr Conduction Heart Cell 169:510–522e20. 10.1016/j.cell.2017.03.050 10.1016/j.cell.2017.03.050 PMC5474950 28431249

[84] Ngwenyama N, Kaur K, Bugg D, Theall B, Aronovitz M, Berland R, Panagiotidou S, Genco C, Perrin MA, Davis J, Alcaide P (2022) Antigen presentation by cardiac fibroblasts promotes cardiac dysfunction. Nat Cardiovasc Res 1:761–774. 10.1038/s44161-022-00116-7 36092510 10.1038/s44161-022-00116-7 PMC9451034

[85] Tomasek JJ, Gabbiani G, Hinz B, Chaponnier C, Brown RA (2002) Myofibroblasts and mechano-regulation of connective tissue remodelling. Nat Rev Mol Cell Biol 3:349–363. 10.1038/nrm809 11988769 10.1038/nrm809

[86] Bradshaw AD, DeLeon-Pennell KY (2020) T-cell regulation of fibroblast and cardiac fibrosis. Matrix Biol 91–92. 10.1016/j.matbio.2020.04.001 10.1016/j.matbio.2020.04.001 PMC7434661 32438054

[87] Waickman AT, Powell JD (2012) mTOR, metabolism, and the regulation of T-cell differentiation and function. Immunol Rev 249:43–58. 10.1111/j.1600-065X.2012.01152.x 22889214 10.1111/j.1600-065X.2012.01152.x PMC3419491

[88] Linke M, Fritsch SD, Sukhbaatar N, Hengstschläger M, Weichhart T (2017) mTORC1 and mTORC2 as regulators of cell metabolism in immunity. FEBS Lett 591:3089–3103. 10.1002/1873-3468.12711 28600802 10.1002/1873-3468.12711 PMC6322652

[89] Su T, Huang K, Mathews KG, Scharf VF, Hu S, Li Z, Frame BN, Cores J, Dinh P-U, Daniele MA, Ligler FS, Cheng K (2020) Cardiac stromal cell Patch Integrated with Engineered Microvessels improves recovery from myocardial infarction in rats and pigs, ACS Biomater. Sci Eng 6:6309–6320. 10.1021/acsbiomaterials.0c00942 10.1021/acsbiomaterials.0c00942 PMC8285028 33449654

[90] Reus TL, Robert AW, Da Costa MBA, de Aguiar AM, Stimamiglio MA (2016) Secretome from resident cardiac stromal cells stimulates proliferation, cardiomyogenesis and angiogenesis of progenitor cells. Int J Cardiol 221:396–403. 10.1016/j.ijcard.2016.06.199 27404713 10.1016/j.ijcard.2016.06.199

[91] Rossini A, Frati C, Lagrasta C, Graiani G, Scopece A, Cavalli S, Musso E, Baccarin M, Di Segni M, Fagnoni F, Germani A, Quaini E, Mayr M, Xu Q, Barbuti A, DiFrancesco D, Pompilio G, Quaini F, Gaetano C, Capogrossi MC (2011) Human cardiac and bone marrow stromal cells exhibit distinctive properties related to their origin. Cardiovascular Res 89:650–660. 10.1093/cvr/cvq290 10.1093/cvr/cvq290 20833652

[92] Bolli R, Tang X-L, Sanganalmath SK, Rimoldi O, Mosna F, Abdel-Latif A, Jneid H, Rota M, Leri A, Kajstura J (2013) Intracoronary Delivery of Autologous Cardiac Stem cells improves cardiac function in a Porcine Model of Chronic ischemic cardiomyopathy. Circulation 128. 10.1161/CIRCULATIONAHA.112.001075 10.1161/CIRCULATIONAHA.112.001075 PMC3807652 23757309

[93] Hu X, Dai S, Wu W-J, Tan W, Zhu X, Mu J, Guo Y, Bolli R, Rokosh G (2007) Stromal cell–derived Factor-1α confers Protection Against Myocardial Ischemia/Reperfusion Injury. Circulation 116:654–663. 10.1161/CIRCULATIONAHA.106.672451 17646584 10.1161/CIRCULATIONAHA.106.672451 PMC3640445

[94] Golpanian S, Wolf A, Hatzistergos KE, Hare JM (2016) Rebuilding the damaged heart: mesenchymal stem cells, cell-based therapy, and Engineered Heart tissue. Physiol Rev 96:1127–1168. 10.1152/physrev.00019.2015 27335447 10.1152/physrev.00019.2015 PMC6345247

[95] Sagaradze GD, Basalova NA, Efimenko AY, Tkachuk VA (2020) Mesenchymal Stromal Cells as Critical Contributors to Tissue Regeneration, Frontiers in Cell and Developmental Biology 8 https://www.frontiersin.org/articles/10.3389/fcell.2020.576176 (accessed May 22, 2023) 10.3389/fcell.2020.576176 PMC7546871 33102483

[96] Cho D-I, Kim MR, Jeong H, Jeong HC, Jeong MH, Yoon SH, Kim YS, Ahn Y (2014) Mesenchymal stem cells reciprocally regulate the M1/M2 balance in mouse bone marrow-derived macrophages. Exp Mol Med 46:e70–e70. 10.1038/emm.2013.135 24406319 10.1038/emm.2013.135 PMC3909888

[97] Wolfien M, Klatt D, Salybekov AA, Ii M, Komatsu-Horii M, Gaebel R, Philippou-Massier J, Schrinner E, Akimaru H, Akimaru E, David R, Garbade J, Gummert J, Haverich A, Hennig H, Iwasaki H, Kaminski A, Kawamoto A, Klopsch C, Kowallick JT, Krebs S, Nesteruk J, Reichenspurner H, Ritter C, Stamm C, Tani-Yokoyama A, Blum H, Wolkenhauer O, Schambach A, Asahara T, Steinhoff G (2020) Hematopoietic stem-cell senescence and myocardial repair - coronary artery disease genotype/phenotype analysis of post-MI myocardial regeneration response induced by CABG/CD133 + bone marrow hematopoietic stem cell treatment in RCT PERFECT phase 3. EBioMedicine 57:102862. 10.1016/j.ebiom.2020.102862 32629392 10.1016/j.ebiom.2020.102862 PMC7339012

[98] Kim S-S, Lim S-H, Cho SW, Gwak S-J, Hong Y-S, Chang BC, Park MH, Song KW, Choi CY, Kim B-S (2006) Tissue engineering of heart valves by recellularization of glutaraldehyde-fixed porcine valves using bone marrow-derived cells. Exp Mol Med 38:273–283. 10.1038/emm.2006.33 16819286 10.1038/emm.2006.33

[99] Assmus B, Leistner DM, Schächinger V, Erbs S, Elsässer A, Haberbosch W, Hambrecht R, Sedding D, Yu J, Corti R, Mathey DG, Barth C, Mayer-Wehrstein C, Burck I, Sueselbeck T, Dill T, Hamm CW, Tonn T, Dimmeler S, Zeiher AM, for the REPAIR-AMI Study Group, Estel S, Braun H, Geweyer I, Palapies L (2014) Long-term clinical outcome after intracoronary application of bone marrow-derived mononuclear cells for acute myocardial infarction: migratory capacity of administered cells determines event-free survival. Eur Heart J 35:1275–1283. 10.1093/eurheartj/ehu062 24569031 10.1093/eurheartj/ehu062

[100] Méndez-Barbero N, Gutiérrez-Muñoz C, Colio LMB (2021) Cellular Crosstalk between endothelial and smooth muscle cells in Vascular Wall Remodeling. Int J Mol Sci 22:7284. 10.3390/ijms22147284 34298897 10.3390/ijms22147284 PMC8306829

[101] Nakagami H, Maeda K, Morishita R, Iguchi S, Nishikawa T, Takami Y, Kikuchi Y, Saito Y, Tamai K, Ogihara T, Kaneda Y (2005) Novel autologous cell therapy in ischemic limb disease through growth factor secretion by cultured adipose tissue–derived stromal cells, arteriosclerosis, thrombosis, and Vascular Biology 25. 2542–2547. 10.1161/01.ATV.0000190701.92007.6d 10.1161/01.ATV.0000190701.92007.6d 16224047

[102] Díaz-Díaz C, Fernandez de Manuel L, Jimenez-Carretero D, Montoya MC, Clavería C, Torres M (2017) Pluripotency surveillance by myc-driven competitive elimination of differentiating cells. Dev Cell 42:585–599e4. 10.1016/j.devcel.2017.08.011 28919206 10.1016/j.devcel.2017.08.011

[103] Merino MM, Rhiner C, Lopez-Gay JM, Buechel D, Hauert B, Moreno E (2015) Elimination of Unfit Cells Maintains Tissue Health and Prolongs Lifespan. Cell 160:461–476. 10.1016/j.cell.2014.12.017 25601460 10.1016/j.cell.2014.12.017 PMC4313366

[104] Leri A, Anversa P (2013) Stem cells and myocardial regeneration. Circulation 127:165–168. 10.1161/CIRCULATIONAHA.112.153973 23224060 10.1161/CIRCULATIONAHA.112.153973 PMC3551577

[105] Fort L, Gama V, Macara IG (2022) Stem cell conversion to the cardiac lineage requires nucleotide signalling from apoptosing cells. Nat Cell Biol 24:434–447. 10.1038/s41556-022-00888-x 35414019 10.1038/s41556-022-00888-x PMC9054036

[106] Pronobis MI, Poss KD (2020) Signals for cardiomyocyte proliferation during zebrafish heart regeneration. Curr Opin Physiol 14:78–85. 10.1016/j.cophys.2020.02.002 32368708 10.1016/j.cophys.2020.02.002 PMC7197729

[107] Hurwitz SN, Jung SK, Kurre P (2020) Hematopoietic stem and progenitor cell signaling in the niche. Leukemia 34:3136–3148. 10.1038/s41375-020-01062-8 33077865 10.1038/s41375-020-01062-8

[108] Lu L-F, Thai T-H, Calado DP, Chaudhry A, Kubo M, Tanaka K, Loeb GB, Lee H, Yoshimura A, Rajewsky K, Rudensky AY (2009) Foxp3-dependent microRNA155 confers competitive fitness to regulatory T cells through targeting SOCS1. Immunity 30:80–91. 10.1016/j.immuni.2008.11.010 19144316 10.1016/j.immuni.2008.11.010 PMC2654249

[109] Leri A, Anversa P (2013) Stem cells and myocardial regeneration: Cooperation wins over competition. Circulation 127:165–168. 10.1161/CIRCULATIONAHA.112.153973 23224060 10.1161/CIRCULATIONAHA.112.153973 PMC3551577

